# Smoking cessation and bronchial epithelial remodelling in COPD: a cross-sectional study

**DOI:** 10.1186/1465-9921-8-85

**Published:** 2007-11-26

**Authors:** Thérèse S Lapperre, Jacob K Sont, Annemarie van Schadewijk, Margot ME Gosman, Dirkje S Postma, Ingeborg M Bajema, Wim Timens, Thais Mauad, Pieter S Hiemstra, the GLUCOLD Study Group

**Affiliations:** 1 Dept. of Pulmonology, Leiden University Medical Centre, Leiden, The Netherlands; 2 Dept. of Medical Decision Making, Leiden University Medical Centre, Leiden, The Netherlands; 3 Dept. of Pathology, Leiden University Medical Centre, Leiden, The Netherlands; 4 Dept. of Pathology, University of Sao Paulo, Sao Paulo, Brazil; 5 Dept. of Pulmonology, University Medical Centre Groningen, The Netherlands; 6 Dept. of Pathology, University Medical Centre Groningen, The Netherlands; 7 The GLUCOLD Study Group: Groningen Leiden Universities and Corticosteroids in Obstructive Lung Disease, a full list of members is listed at the back

## Abstract

**Background:**

Chronic Obstructive Pulmonary Disease (COPD) is associated with bronchial epithelial changes, including squamous cell metaplasia and goblet cell hyperplasia. These features are partially attributed to activation of the epidermal growth factor receptor (EGFR). Whereas smoking cessation reduces respiratory symptoms and lung function decline in COPD, inflammation persists. We determined epithelial proliferation and composition in bronchial biopsies from current and ex-smokers with COPD, and its relation to duration of smoking cessation.

**Methods:**

114 COPD patients were studied cross-sectionally: 99 males/15 females, age 62 ± 8 years, median 42 pack-years, no corticosteroids, current (n = 72) or ex-smokers (n = 42, median cessation duration 3.5 years), postbronchodilator FEV_1 _63 ± 9% predicted. Squamous cell metaplasia (%), goblet cell (PAS/Alcian Blue^+^) area (%), proliferating (Ki-67^+^) cell numbers (/mm basement membrane), and EGFR expression (%) were measured in intact epithelium of bronchial biopsies.

**Results:**

Ex-smokers with COPD had significantly less epithelial squamous cell metaplasia, proliferating cell numbers, and a trend towards reduced goblet cell area than current smokers with COPD (p = 0.025, p = 0.001, p = 0.081, respectively), but no significant difference in EGFR expression. Epithelial features were not different between short-term quitters (<3.5 years) and current smokers. Long-term quitters (≥3.5 years) had less goblet cell area than both current smokers and short-term quitters (medians: 7.9% vs. 14.4%, p = 0.005; 7.9% vs. 13.5%, p = 0.008; respectively), and less proliferating cell numbers than current smokers (2.8% vs. 18.6%, p < 0.001).

**Conclusion:**

Ex-smokers with COPD had less bronchial epithelial remodelling than current smokers, which was only observed after long-term smoking cessation (>3.5 years).

**Trial registration:**

NCT00158847

## Background

Chronic Obstructive Pulmonary Disease (COPD) is defined by progressive airflow limitation and airway inflammation [1], caused predominantly by cigarette smoking. Additionally, the airway epithelium undergoes alterations, including squamous cell metaplasia, goblet and basal cell hyperplasia [2]. These findings are important for our understanding of the pathogenesis of COPD, since bronchial epithelial cells orchestrate an adequate maintenance of lung homeostasis by mucus production, ciliary beating, secretion of antimicrobial products and adequate immunological drive in response to noxious stimuli. Goblet cell hyperplasia is more pronounced in smokers with COPD compared to those without, suggesting a role in the development of airflow limitation [3]. In addition, it contributes to mucus hypersecretion, which is associated with morbidity and mortality in COPD [4,5]. Squamous cell metaplasia impairs mucociliary clearance and contributes to the increased risk of squamous cell carcinoma as observed in COPD [6].

The mechanisms underlying epithelial alterations in COPD are incompletely understood. The epidermal growth factor receptor (EGFR) cascade has been shown to be involved in mucin production and goblet cell hyperplasia [7,8], repair of damaged epithelium [7,8], as well as development of squamous cell carcinoma [9]. A wide variety of stimuli can induce EGFR activation *in vitro *and in animals, including cigarette smoke [7,8]. Additionally, epithelial EGFR expression is increased in bronchial biopsies from smokers with [10,11] and without [11,12] COPD compared to non-smokers. Previously, we have observed higher epithelial EGFR expression in ex-smokers with COPD compared to non-COPD, but not in current smokers, suggesting that current smoking may obscure differences in EGFR expression [13]. Therefore, EGFR activation may play a role in epithelial phenotypic alterations observed in COPD through active smoking.

Smoking cessation improves respiratory symptoms and lung function decline in COPD, mostly within the first year after cessation [14,15], but interestingly bronchial airway inflammation persists or even worsens [16,17]. To our knowledge, there are no studies comparing bronchial epithelial features between current and ex-smokers with established COPD. Possibly, smoking cessation contributes to restoration of epithelial characteristics in the large airways of COPD patients, which are directly and continuously exposed to the noxious substances present in cigarette smoke, thereby contributing to the clinical benefits observed after smoking cessation. Therefore, it needs to be addressed whether bronchial epithelial alterations and EGFR expression in large airways are reversible with smoking cessation and related to the duration of smoking cessation in COPD.

We hypothesised that bronchial epithelial cell proliferation and differentiation in patients with COPD is more pronounced in active smokers than in those who stopped smoking, and that this difference is influenced by the duration of smoking cessation. Additionally, we questioned whether the epithelial changes are associated with EGFR expression. We therefore investigated the extent of epithelial goblet cell hyperplasia, proliferation, squamous cell metaplasia, and EGFR expression in bronchial biopsies of current and ex-smokers with established COPD in a large cross-sectional study.

## Methods

### Subjects

114 patients with COPD, who participated in a two-centre trial (Groningen Leiden Universities and Corticosteroids in Obstructive Lung Disease; GLUCOLD study), were included in this cross-sectional study. Patient characteristics and methods have been described in detail previously [17,18]. In brief, all patients had irreversible airflow limitation [postbronchodilator FEV_1 _and FEV_1_/IVC < 90% *confidence interval *(CI) of predicted value] and chronic respiratory symptoms, they were all current or ex-smokers (quit = 1 year), with at least 10 pack-years of smoking. Patients did not use a course of steroids during the last three months, and did not have maintenance treatment with inhaled or oral steroids during the last six months. They were allowed to use short-acting bronchodilators, and were in clinical stable condition. The medical ethics committees of each centre approved the study and all patients gave their written informed consent.

### Lung function

Spirometry, reversibility to salbutamol, and diffusing capacity were measured according to previously described methods in order to characterise the patients [18].

### Bronchoscopy

Fiberoptic bronchoscopy was performed using a standardised protocol according to recent recommendations [19] as described previously [17]. Smokers were requested to refrain from smoking on the day of the bronchoscopy. Patients received premedication (400 μg salbutamol p.i., 20 mg codeine p.o., 0.5 mg atropine s.c.) and local anaesthesia (lidocaine). The bronchoscopies were performed by experienced pulmonary physicians using a fiberoptic bronchoscope (18×, outer diameter 6 mm, Pentax Optical Co., Japan) and pairs of cup forceps (Reda, Tuttlingen, Germany). Six macroscopically adequate bronchial biopsy specimens were taken from (sub) segmental carinae in the right or left lower lobe.

### Biopsy processing and staining

Four paraffin-embedded biopsies were cut in 4 μm thick sections and haematoxylin/eosin staining was used for evaluation and selection of the two morphological best biopsies per patient for analysis (without crushing artefacts, large blood clots, or only epithelial scrapings). Sections were stained with Periodic acid-Schiff/Alcian blue (PAS/AB) and counterstained with Nuclear Fast Red to identify all secretory cells. Immunohistochemistry was performed as described previously for inflammatory cells [17], using specific antibodies against Ki-67 as a marker of proliferation (Dako M7240, dilution 1:100), and EGFR (Biogenex nr MU207-UC, dilution 1:50). Antigen retrieval was obtained using citrate for Ki-67 and pepsin for EGFR.

### Analysis of bronchial biopsies

Total biopsy images were prepared with a 3-chip colour camera and analysed by means of image analysis software (Zeiss Vision KS400 system, Carl Zeiss, Göttingen, Germany) as follows. First, the length of the basement membrane was traced of all intact non-squamous metaplastic epithelium (A), intact squamous metaplastic epithelium (B), and damaged epithelium (C) in PAS/AB stained sections, in order to calculate the % intact epithelium [(A+B)/(A+B+C)] and the % metaplastic epithelium [B/(A+B)]. In addition, the presence of metaplastic epithelium was also scored as absent (0) or present (1). Intact epithelium (A+B) was defined as a layer of both basal and columnar cells without detachment from the basement membrane, including areas of goblet cell hyperplasia or squamous metaplasia [20]. Consequently, damaged epithelium (C) was defined as all remaining epithelium, including denuded basement membrane. Squamous cell metaplasia was defined as pseudostratisfied multilayered epithelium consisting of polygonal cells covered by flattened layer of squamous cells and absence of ciliated cells [21]. Subsequently, the number of Ki-67 positively staining cells was counted in intact epithelium by a validated full automated procedure [22], and expressed as the number of Ki-67^+ ^cells/mm basement membrane. Densitometric analysis of PAS/AB and EGFR in intact epithelium (A+B) was also performed fully automated as follows [22]. A linear combination of Red-, and Blue-filtered greyscale images was used, in order to derive a greyscale image (range 0–255) in which the "purple" staining (PAS/AB) and the "brown-red" staining (EGFR) highlighted above background. Results were expressed as the percentage of intact epithelium stained by PAS/AB and EGFR. In addition, EGFR staining intensity of positive areas was expressed as the average greyvalue, after normalization of the distribution towards the background peak (white: greyvalue 255) and subsequent inversion of the greyvalue distribution. Mean values of two biopsies analysed per patient were used for analysis.

### Statistical analysis

Mean values and standard deviations (SD) were computed and presented, or median with interquartile range (IQR) in case of non-normal distributed variables. Since most epithelial markers were still non-normal distributed after log-transformation, these data were analysed using non-parametric tests. Differences between smokers and ex-smokers were explored using Chi-square tests or 2-tailed unpaired t-tests for patient characteristics, and Mann Whitney tests for epithelial features. To study the association with duration of smoking cessation, we compared smokers with ex-smokers who quit <3.5 years and those who quit ≥3.5 years ago, since this was the median duration of smoking cessation, using Kruskal-Wallis tests. If these were significant, Mann Whitney tests were applied for further exploration of between-group differences. Univariate correlations were evaluated using Spearman rank correlation coefficient. To analyse correlations with years since smoking cessation, current smokers were included in the analysis as 0 years stopped. Multivariate linear regression analysis was applied to adjust for significant differences in patient characteristics between the groups, such as age, pack-years, and FEV_1_/IVC. PAS/AB^+ ^area was measured in total intact epithelium, including squamous cell metaplasia, which by definition does not contain goblet cells. Therefore, linear regression analysis was also applied to adjust for % squamous cell metaplasia when analysing PAS/AB^+ ^differences between groups. SPSS 12.0 (SPSS Inc., Chicago, IL) software was used for statistical analysis.

## Results

### Patient characteristics

Patient characteristics of the 114 patients and subgroups of smokers and ex-smokers have been described in detail previously [17,18] (Table 1). Patients had moderate to severe COPD, based on a postbronchodilator FEV_1 _of 63.0 (8.8)% predicted, and a median smoking history of 42 pack-years. Of the 114 COPD patients included in this study, 72 were current smokers and 42 were ex-smokers. Median duration of smoking cessation in ex-smokers was 3.5 years (IQR: 1–10 years). Differences in patient characteristics between the groups are presented in table 1.

**Table 1:** Patient characteristics

	COPD Current smokers	COPD Ex-smokers		
		*combined group*	*quit < 3.5 yrs*	*quit ≥3.5 yrs*
General				
Sex (M/F, n)	59/13	40/2 *	20/1	20/1
Age (yrs)	60 ± 8	64 ± 7 *	61 ± 8	67 ± 4 *†
Pack-years (yrs)	43 (32–56)	37 (28–53)	45 (29–65)	35 (26–41) *
Duration of smoking cessation (yrs)	-	3.5 (1–10)	1.0 (1.0–2.0)	10 (6.5–14.5)
Smoking duration (yrs)	44 ± 8	41 ± 10	43 ± 11	39 ± 8
Chronic bronchitis (%)	55.6	31.0 *	23.8 *	38.1
*Lung Function*				
Postbronchodilator FEV_1 _(%pred)	63.3 ± 8.3	62.5 ± 9.6	62.6 ± 10	62.5 ± 9.4
Postbronchodilator FEV_1_/IVC (%)	49.5 ± 8.5	46.0 ± 8.3 *	45.3 ± 8.6 *	46.7 ± 8.1
ΔFEV_1 _(%pred)	6.9 ± 5.2	6.8 ± 4.5	6.9 ± 3.9	6.8 ± 5.1
K_CO _(%pred)	73.3 ± 25.1	80.4 ± 25.9	75.3 ± 24.9	85.7 ± 26.5 *

Data are presented as mean ± standard deviation or median (IQR: 25^th ^– 75^th ^percentile), ex-smokers are divided in two groups based on median duration of smoking cessation (3.5 years). FEV_1 _= Forced expiratory volume in one second, IVC = Inspiratory vital capacity, ΔFEV_1 _= Reversibility to salbutamol (change in FEV_1 _as percentage of predicted), K_CO _= Diffusing capacity for carbon monoxide per liter alveolar volume, pred = predicted. Patient characteristics have been described before [17].

* p < 0.05: compared to COPD smokers [Chi-square tests for sex differences, 2-tailed unpaired t-tests for other (log-transformed) data]; † p < 0.05: compared to COPD ex-smokers who quit <3.5 yrs (2-tailed unpaired t-tests)

### Epithelial features in smokers versus ex-smokers with COPD

All 114 patients underwent bronchoscopy; from one patient (ex-smoker) none of the biopsies taken were adequate for analysis. The median analysed basement membrane length per biopsy was 5.03 (IQR: 3.64–6.14) mm, of which 1.12 (0.59–2.11) mm was intact epithelium. Characteristics of intact epithelium in the total group of patients were: 9.74 (3.54–34.0) Ki-67^+ ^cells/mm BSM, 0 (0–19.7)% squamous cell metaplasia (37.3% of patients showed squamous cell metaplasia), 12.4 (4.69–18.9)% PAS/AB^+ ^area, and 10.4 (3.25–18.9)% EGFR^+ ^area.

Ex-smokers had significantly less Ki-67^+ ^cell numbers, and % of patients with squamous cell metaplasia as well as % of epithelium with squamous cell metaplasia (p = 0.001, p = 0.016, p = 0.025; respectively, Table 2) than current smokers. PAS/AB^+ ^area also tended to be lower in ex-smokers, but this was not statistically significant (p = 0.081, Table 2). When adjusting for the presence of squamous metaplasia (which by definition does not contain goblet cells), the difference in PAS/AB^+ ^area became significant (p = 0.014). When differences in sex, age, and FEV_1_/IVC were taken into account in multivariate linear and logistic regression analyses, all epithelial differences remained significant, except for PAS/AB^+ ^area. Epithelial EGFR^+ ^areas and intensities of positive areas showed no differences between smokers and ex-smokers with COPD (Table 2).

**Table 2:** Bronchial epithelial features of smokers and ex-smokers with COPD

	COPD Smokers	COPD Ex-smokers			p-value §
		*combined group*	*quit < 3.5 years*	*quit ≥3.5 years*	
SCM (% of epithelium)	0 (0–27.5)	0 (0-0)*	0 (0–10.4)	0 (0-0)	0.076
SCM (% of patients)	45.7	22.5*	26.3	19.0*	0.049
PAS/AB^+ ^area (%)	14.4 (5.2–20.7)	8.1 (3.7–17.2)†	13.5 (6.6–19.6)	7.9 (2.2–16.2)*‡	0.011
Ki-67^+ ^cells (/mm BSM)	18.6 (5.3–38.8)	6.2 (1.5–15.6)*	6.9 (4.4–27.6)	2.8 (0.23–13.1)*	<0.001
EGFR^+ ^area (%)	11.4 (3.2–17.6)	8.2 (3.2–20.4)	6.7 (2.4–20.4)	8.6 (3.9–21.5)	0.95
Intensity EGFR^+ ^area (grey value)	697 (175–1182)	513 (203–1479)	372 (125–1479)	517 (215–1424)	0.95

Data represent median (IQR: 25^th ^– 75^th ^percentile). PAS/AB = Periodic acid-Schiff/Alcian blue, SCM = squamous cell metaplasia, EGFR = Epidermal growth factor receptor.

* p < 0.05: compared to COPD smokers, † p < 0.05: compared to COPD smokers adjusted for differences in squamous cell metaplasia (linear regression), ‡ p < 0.05: compared to COPD ex-smokers who quit <3.5 yrs, §p-value from Kruskal-Wallis test between current smokers, < and ≥3.5 yrs ex-smokers with COPD.

### Duration of smoking cessation and epithelial features in COPD

**Figure 1:**
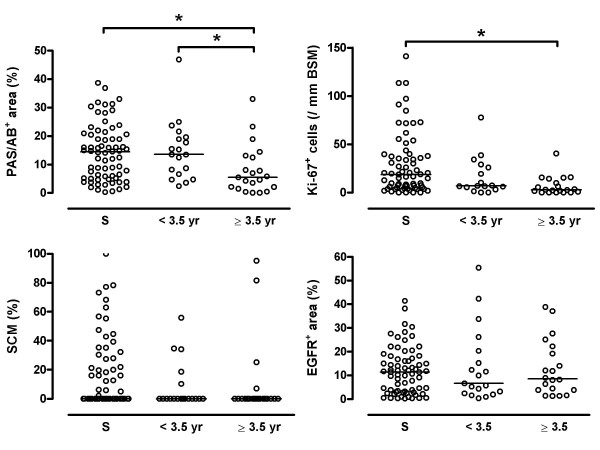
**Epithelial phenotype in smokers (S) and ex-smokers with COPD**. Individual values of: (A) % PAS/AB^+ ^area of intact epithelium, (B) Ki-67^+ ^cells (/mm basement membrane = BSM) in intact epithelium, (C) % squamous cell metaplasia (SCM) of intact epithelium (note: in a large % of patients no SCM was observed), (D) % EGFR^+ ^area of intact epithelium. Data are grouped by COPD smokers (S), COPD ex-smokers who quit < 3.5 years ago (<3.5 yr), and who quit ≥3.5 years ago (≥3.5 yr). Horizontal bars represent median values, * p < 0.05.

Ki-67^+ ^cell numbers, the presence of squamous cell metaplasia, and the % PAS/AB^+ ^area, were different between current smokers, ex-smokers who quit <3.5 years ago, and ex-smokers who quit ≥3.5 years ago (Kruskal Wallis: p = 0.011, p < 0.001, p = 0.049; respectively, Table 2, Figure 1). Percentage squamous cell metaplasia and EGFR^+ ^areas and intensities were not significantly different between these three groups (Table 2, Figure 1). There were no differences between current smokers and those who quit <3.5 years ago for any of the epithelial features. In contrast, those who quit ≥3.5 years ago had significantly less Ki-67^+ ^cell numbers, presence of squamous cell metaplasia, and % PAS/AB^+ ^area than current smokers (p < 0.001, p = 0.029, p = 0.005, respectively; Table 2, Figure 1). The differences in PAS/AB and Ki-67 remained significant when adjusting for age and pack-year differences between both groups. Moreover, the % PAS/AB^+ ^area was also lower in long-term quitters than those who quit <3.5 years ago (p = 0.008), and tended to be lower for Ki-67^+ ^cell numbers (p = 0.050). When adjusting for differences in age, PAS/AB significance was lost (p = 0.061).

There was a significant inverse relationship between the duration of smoking cessation (including current smokers as 0 years stopped) and Ki-67^+ ^cell numbers (r_s _= -0.354, p < 0.001), % squamous cell metaplasia (r_s _= -0.212, p = 0.004), and % PAS/AB^+ ^area (r_s _= -0.235; p = 0.013), but not with EGFR expression.

### Relation of epithelial features with smoking, symptoms and lung function

The duration of smoking tended to be associated with the number of Ki-67^+ ^cells (r_s _= 0.180, p = 0.065), whereas the number of pack-years smoked was not associated with epithelial features. 46.5% of all patients reported symptoms of chronic bronchitis, and although ex-smokers had significantly less symptoms of chronic bronchitis (Table 1) and % of PAS/AB^+ ^area, there was no relation between the presence of these symptoms and the % of PAS/AB^+ ^area (p = 0.78). Epithelial features were not associated with the degree of airflow limitation.

### Relation between epithelial cell proliferation and differentiation

Ki-67^+ ^cell numbers and the % squamous cell metaplasia were positively associated with one another (r_s _= 0.586, p < 0.001). Finally, Ki-67^+ ^cell numbers were also positively associated with % EGFR^+ ^area (r_s _= 0.210, p = 0.031).

## Discussion

In the present study, we demonstrated that long-term ex-smokers with COPD had less bronchial epithelial mucin stores, proliferating cells, and squamous cell metaplasia than current smokers with COPD. Moreover, these epithelial differences in ex-smokers were only significant after a long-term period of smoking cessation (>3.5 years). In contrast, epithelial EGFR expression was not different between current and ex-smokers with COPD. These results may indicate that smoking cessation reverses smoking-induced bronchial epithelial cell proliferation and differentiation in patients with COPD, and the magnitude of this effect increases with longer duration of smoking cessation. In addition, our results suggest that these smoking cessation-induced epithelial changes in COPD are not accompanied by reduced EGFR expression.

Our observation of lower bronchial epithelial mucin stores, proliferating cells, and squamous cell metaplasia in large airways of ex-smokers as compared to current smokers with COPD, and the association with duration of cessation, is novel. These results are in contrast to other, smaller studies showing no differences in epithelial features in ex-smokers compared to smokers both with and without COPD [21,23]. However, the finding that ex-smokers with chronic bronchitis (with or without airflow limitation), had less goblet cell metaplasia in small airways than current smokers [24] is in line with our findings in COPD patients. The effect of smoking cessation and duration of cessation on squamous cell metaplasia and proliferation has been examined previously in bronchial biopsies [25]. Although it was not mentioned whether these patients had respiratory symptoms and/or COPD, the latter study also reported less squamous cell metaplasia and epithelial proliferation in ex-smokers. Our result of similar EGFR expression in ex-smokers compared to current smokers with COPD, is also novel and in contrast with observations in smokers without COPD, where lower bronchial EGFR expression was observed in ex-smokers [12]. Taken together, it can now be inferred that smoking cessation results in decreased epithelial mucin stores, proliferation, and squamous cell metaplasia, in large airways of patients with COPD, but that it does not affect EGFR expression.

There are a few important considerations when interpreting our results. We included a large (n = 114) group of well-characterised patients with stable COPD, not inhaling steroids for at least six months or oral steroids for at least three months, and without a clinical diagnosis of asthma. First, it needs to be emphasised that this was a cross-sectional study, and it cannot be ruled out that our ex-smoking group is a selected group of patients who quit smoking because they suffered more from smoking related symptoms, and may already have had a different epithelial morphology before quitting. Yet, in the present study ex-smokers had significantly less respiratory symptoms than current smokers, whilst having similar pack-years and duration of smoking. In addition, we also reported analyses adjusted for clinical differences between the groups (sex, age, FEV_1_/IVC, pack-years). Second, comparable to previously published cross-sectional studies we did not confirm smoking status by laboratory tests, and therefore cannot rule out that some ex-smokers were still smoking. Third, our definitions of intact epithelium and squamous cell metaplasia were very strict, which could have led to an underestimation. Fourth, we cannot exclude the possibility that mechanical injury induced during bronchoscopy may have interfered with our analyses of epithelial damage. Fifth, we applied fully automated image analysis for quantification of cell numbers, positively stained areas, and densitometry analyses, and therefore minimised potential observer biases. Finally, we did not include a control group of smokers without COPD and therefore cannot conclude whether the observed effects of smoking cessation are specific to COPD. Taken together, it seems unlikely that our results are strongly affected by methodological errors.

How can we interpret these data? The (partial) reversibility of mucin stores, squamous cell metaplasia, and proliferation after smoking cessation, supports a causal relationship between cigarette smoke exposure and these epithelial features in COPD. *In vivo*, the proliferative response in smokers may be due to a direct mitogenic effect of cigarette smoke (26), but may also result from chronic inflammation, tissue damage and wound healing [26]. An inadequate repair response to smoke-induced injury may lead to a sustained increase in epithelial proliferation and/or altered differentiation. Increased proliferation may accompany squamous cell metaplasia [21,25], which is in line with our observation that squamous cell metaplasia is related to proliferating cell numbers. This squamous metaplasia may serve to protect the underlying tissue against the injurious effects of cigarette smoke. According to our results, smoking cessation may reverse this process in COPD, at least partially.

The observed decrease in mucin stores in long-term ex-smokers with COPD, and its relation with duration of smoking cessation, can be explained by decreased goblet cell numbers, decreased mucin production, and/or increased mucin secretion. The few previous studies examining goblet cell hyperplasia in relation to smoking cessation in humans used semi-quantitative scoring systems, but not cell numbers [23,24]. Animal studies suggest that a reduction in secretory cell numbers occurs after smoking cessation [27]. The observed decrease in mucin stores in ex-smokers in our study probably reflects a decrease in the major mucin in surface epithelium, MUC5AC [28]. MUC5AC and total mucin in bronchial epithelium are correlated, and both are increased in smokers compared to non-smokers [11]. In addition, small airway epithelial MUC5AC expression is lower in ex-smokers compared to current smokers with and without COPD [29], which is also in line with our conclusion. Although ex-smokers exhibited fewer symptoms of chronic bronchitis and a smaller amount of mucin stores, chronic bronchitis was not associated with mucin stores. This can be explained by the fact that mucin is produced by both goblet cells in the surface epithelium and by submucosal glands [30], whereas these latter were not included in the present analysis. This conclusion is supported by the results of a recent study that also failed to reveal differences in epithelial mucin expression in peripheral airways of COPD patients with or without chronic bronchitis [29]. Finally, it needs to be noted that our study focused on mucin expression and not on secretion, whereas the latter is the main feature of chronic bronchitis.

The changes in epithelial mucin stores, proliferating cells, and squamous cell metaplasia were most pronounced in COPD patients who had stopped smoking more than 3.5 years ago. Correspondingly, inflammation initially persists after smoking cessation [17,31]. This suggests a long-term effect of smoking on bronchial regulatory networks, which is not restored immediately after removing the initial stimulus, i.e. cigarette smoke. In contrast, the greatest improvements in respiratory symptoms and lung function decline occur within the first year after cessation [14,15]. Therefore, other pathological mechanisms that reverse more rapidly after cessation should be involved in these clinical beneficial effects of smoking cessation.

Cigarette smoke causes both mucus hypersecretion and increases the number of goblet cells through activation of the EGFR system [32]. In the present study, there were no differences between current and ex-smokers with COPD in EGFR expression, suggesting that differences in EGFR activation, rather than expression, are present in ex-versus current smokers with COPD. We did not pursue this possibility further, since recent attempts in our laboratory to demonstrate *in situ *EGFR phosphorylation in lung tissue by immunohistochemical methods using phosphospecific antibodies were not successful [13]. Since pro-inflammatory cytokines, such as TNF-α, increase EGFR expression [33], our observation that there is no difference in EGFR expression between ex- and current smokers may be related to the persistence of bronchial inflammation [16,17] in ex-smokers with COPD. The large inter-individual differences in EGFR expression could also explain why there were no differences between the groups in this cross-sectional analysis. Alternatively, EGFR independent pathways may contribute to epithelial mucin production.

What could be the clinical implications of our findings? There is good evidence that smoking cessation results in less respiratory symptoms [14], less decline in FEV_1 _[15], and less severe airway hyperresponsiveness [34,35], whereas inflammation persists [16,17]. Our data suggest that smoking-induced bronchial epithelial goblet cell hyperplasia, proliferation, and squamous cell metaplasia are reduced with long-term smoking cessation. These epithelial features might contribute to COPD by facilitating colonization of the airways by respiratory pathogens, secondary to loss of cilia, increased mucus secretion, and epithelial injury [36]. The chronic colonization of the airways may enhance airway inflammation and further epithelial injury. In addition, mucus hypersecretion may cause airways obstruction in peripheral airways [37]. Reversal of epithelial remodelling may therefore contribute to reduced progression of COPD attributable to restored mucociliary clearance, resulting in reduced respiratory colonization [38] and exacerbations, and less small airways obstruction. In addition, reduced epithelial proliferation and squamous cell metaplasia in ex-smokers with COPD may decrease the risk of squamous cell carcinoma development. Whether the observed (partial) reversibility of epithelial remodelling is associated with the clinical benefits of smoking cessation in patients with COPD, remains to be established in longitudinal studies.

## Conclusion

The present study has shown that ex-smokers with COPD have less bronchial epithelial mucin stores, proliferating cells, and squamous cell metaplasia than current smokers with COPD, whereas epithelial EGFR expression was not different between both groups. These epithelial changes in ex-smokers were more pronounced with longer duration of smoking cessation, and significant after 3.5 years smoking cessation. This suggests that the clinical benefits of smoking cessation in COPD patients may be in part attributable to a restoration of epithelial homeostasis.

## Competing interests

The author(s) declare that they have no competing interests.

## Authors' contributions

TL recruited and characterised patients, coordinated the study, performed tissue and statistical analyses, and drafted the manuscript; JS participated in the image analysis, and helped drafting the manuscript; AS performed tissue analyses; MG recruited patients and characterised, coordinated the study, and helped drafting the manuscript; DP participated in the design of the study, coordination of the study, and helped drafting the manuscript; IB participated in pathological analysis, and helped drafting the manuscript; WT participated in the design of the study, coordination of the study, participated in pathological analysis, and helped drafting the manuscript; TM participated in pathological analysis, and helped drafting the manuscript; PH participated in the design of the study, coordination of the study, participated in pathological analysis, and helped drafting the manuscript. All authors read and approved the final manuscript.

## References

[B1] Global Initiative for Chronic Obstructive Lung Disease (2006). Global strategy for the Diagnosis, Management and Prevention of Chronic Obstructive Pulmonary Disease. NHLBI/WHO workshop report. Available at: www goldcopd com.

[B2] Jeffery PK (2000). Comparison of the structural and inflammatory features of COPD and asthma. Giles F. Filley Lecture. Chest.

[B3] Innes AL, Woodruff PG, Ferrando RE, Donnelly S, Dolganov GM, Lazarus SC, Fahy JV (2006). Epithelial mucin stores are increased in the large airways of smokers with airflow obstruction. Chest.

[B4] Vestbo J, Prescott E, Lange P (1996). Association of chronic mucus hypersecretion with FEV1 decline and chronic obstructive pulmonary disease morbidity. Copenhagen City Heart Study Group. Am J Respir Crit Care Med.

[B5] Prescott E, Lange P, Vestbo J (1995). Chronic mucus hypersecretion in COPD and death from pulmonary infection. Eur Respir J.

[B6] Papi A, Casoni G, Caramori G, Guzzinati I, Boschetto P, Ravenna F, Calia N, Petruzzelli S, Corbetta L, Cavallesco G, Forini E, Saetta M, Ciaccia A, Fabbri LM (2004). COPD increases the risk of squamous histological subtype in smokers who develop non-small cell lung carcinoma. Thorax.

[B7] Burgel PR, Nadel JA (2004). Roles of epidermal growth factor receptor activation in epithelial cell repair and mucin production in airway epithelium. Thorax.

[B8] Rogers DF (2003). The airway goblet cell. Int J Biochem Cell Biol.

[B9] Franklin WA, Veve R, Hirsch FR, Helfrich BA, Bunn PA (2002). Epidermal growth factor receptor family in lung cancer and premalignancy. Semin Oncol.

[B10] Nadel JA, Burgel PR (2001). The role of epidermal growth factor in mucus production. Curr Opin Pharmacol.

[B11] O'Donnell RA, Richter A, Ward J, Angco G, Mehta A, Rousseau K, Swallow DM, Holgate ST, Djukanovic R, Davies DE, Wilson SJ (2004). Expression of ErbB receptors and mucins in the airways of long term current smokers. Thorax.

[B12] Kurie JM, Shin HJ, Lee JS, Morice RC, Ro JY, Lippman SM, Hittelman WN, Yu R, Lee JJ, Hong WK (1996). Increased epidermal growth factor receptor expression in metaplastic bronchial epithelium. Clin Cancer Res.

[B13] de Boer WI, Hau CM, van Schadewijk A, Stolk J, van Krieken JH, Hiemstra PS (2006). Expression of epidermal growth factors and their receptors in the bronchial epithelium of subjects with chronic obstructive pulmonary disease. Am J Clin Pathol.

[B14] Kanner RE, Connett JE, Williams DE, Buist AS (1999). Effects of randomized assignment to a smoking cessation intervention and changes in smoking habits on respiratory symptoms in smokers with early chronic obstructive pulmonary disease: the Lung Health Study. Am J Med.

[B15] Anthonisen NR, Connett JE, Kiley JP, Altose MD, Bailey WC, Buist AS, Conway WA, Enright PL, Kanner RE, O'Hara P (1994). Effects of smoking intervention and the use of an inhaled anticholinergic bronchodilator on the rate of decline of FEV1. The Lung Health Study. JAMA.

[B16] Rutgers SR, Postma DS, ten Hacken NH, Kauffman HF, van der Mark TW, Koeter GH, Timens W (2000). Ongoing airway inflammation in patients with COPD who do not currently smoke. Thorax.

[B17] Lapperre TS, Postma DS, Gosman MME, Snoeck-Stroband JB, ten Hacken NH, Hiemstra PS, Timens W, Sterk PJ, Mauad T, Group TGS (2006). Relation between duration of smoking cessation and bronchial inflammation in COPD. Thorax.

[B18] Lapperre TS, Snoeck-Stroband JB, Gosman MM, Stolk J, Sont JK, Jansen DF, Kerstjens HA, Postma DS, Sterk PJ, Group TGS (2004). Dissociation of lung function and airway inflammation in chronic obstructive pulmonary disease. Am J Respir Crit Care Med.

[B19] Jeffery P, Holgate S, Wenzel S (2003). Methods for the assessment of endobronchial biopsies in clinical research: application to studies of pathogenesis and the effects of treatment. Am J Respir Crit Care Med.

[B20] Pilette C, Godding V, Kiss R, Delos M, Verbeken E, Decaestecker C, De Paepe K, Vaerman JP, Decramer M, Sibille Y (2001). Reduced Epithelial Expression of Secretory Component in Small Airways Correlates with Airflow Obstruction in Chronic Obstructive Pulmonary Disease. Am J Respir Crit Care Med.

[B21] Aarbiou J, van Schadewijk A, Stolk J, Sont JK, de Boer WI, Rabe KF, van Krieken JH, Mauad T, Hiemstra PS (2004). Human neutrophil defensins and secretory leukocyte proteinase inhibitor in squamous metaplastic epithelium of bronchial airways. Inflamm Res.

[B22] Sont JK, de Boer WI, van Schadewijk WA, Grunberg K, van Krieken JH, Hiemstra PS, Sterk PJ (2003). Fully automated assessment of inflammatory cell counts and cytokine expression in bronchial tissue. Am J Respir Crit Care Med.

[B23] Wright JL, Lawson LM, Pare PD, Wiggs BJ, Kennedy S, Hogg JC (1983). Morphology of peripheral airways in current smokers and ex-smokers. Am Rev Respir Dis.

[B24] Mullen JB, Wright JL, Wiggs BR, Pare PD, Hogg JC (1987). Structure of central airways in current smokers and ex-smokers with and without mucus hypersecretion: relationship to lung function. Thorax.

[B25] Lee JJ, Liu D, Lee JS, Kurie JM, Khuri FR, Ibarguen H, Morice RC, Walsh G, Ro JY, Broxson A, Hong WK, Hittelman WN (2001). Long-term impact of smoking on lung epithelial proliferation in current and former smokers. J Natl Cancer Inst.

[B26] Aarbiou J, Ertmann M, van Wetering S, van Noort P, Rook D, Rabe KF, Litvinov SV, van Krieken JH, de Boer WI, Hiemstra PS (2002). Human neutrophil defensins induce lung epithelial cell proliferation in vitro. J Leukoc Biol.

[B27] Wright JL, Churg A (2002). Smoking cessation decreases the number of metaplastic secretory cells in the small airways of the Guinea pig. Inhal Toxicol.

[B28] Ordonez CL, Khashayar R, Wong HH, Ferrando R, Wu R, Hyde DM, Hotchkiss JA, Zhang Y, Novikov A, Dolganov G, Fahy JV (2001). Mild and moderate asthma is associated with airway goblet cell hyperplasia and abnormalities in mucin gene expression. Am J Respir Crit Care Med.

[B29] Caramori G, Di Gregorio C, Carlstedt I, Casolari P, Guzzinati I, Adcock IM, Barnes PJ, Ciaccia A, Cavallesco G, Chung KF, Papi A (2004). Mucin expression in peripheral airways of patients with chronic obstructive pulmonary disease. Histopathology.

[B30] Rogers DF (2000). Mucus pathophysiology in COPD: differences to asthma, and pharmacotherapy. Monaldi Arch Chest Dis.

[B31] Willemse BW, ten Hacken NH, Rutgers B, Lesman-Leegte IG, Postma DS, Timens W (2005). Effect of 1-year smoking cessation on airway inflammation in COPD and asymptomatic smokers. Eur Respir J.

[B32] Takeyama K, Jung B, Shim JJ, Burgel PR, Dao-Pick T, Ueki IF, Protin U, Kroschel P, Nadel JA (2001). Activation of epidermal growth factor receptors is responsible for mucin synthesis induced by cigarette smoke. Am J Physiol Lung Cell Mol Physiol.

[B33] Schmiegel W, Roeder C, Schmielau J, Rodeck U, Kalthoff H (1993). Tumor necrosis factor alpha induces the expression of transforming growth factor alpha and the epidermal growth factor receptor in human pancreatic cancer cells. Proc Natl Acad Sci U S A.

[B34] Wise RA, Kanner RE, Lindgren P, Connett JE, Altose MD, Enright PL, Tashkin DP (2003). The Effect of Smoking Intervention and an Inhaled Bronchodilator on Airways Reactivity in COPD: The Lung Health Study. Chest.

[B35] Willemse BW, ten Hacken NH, Rutgers B, Lesman-Leegte IG, Timens W, Postma DS (2004). Smoking cessation improves both direct and indirect airway hyperresponsiveness in COPD. Eur Respir J.

[B36] Sethi S, Murphy TF (2001). Bacterial infection in chronic obstructive pulmonary disease in 2000: a state-of-the-art review. Clin Microbiol Rev.

[B37] Hogg JC, Chu F, Utokaparch S, Woods R, Elliott WM, Buzatu L, Cherniack RM, Rogers RM, Sciurba FC, Coxson HO, Pare PD (2004). The nature of small-airway obstruction in chronic obstructive pulmonary disease. N Engl J Med.

[B38] Zalacain R, Sobradillo V, Amilibia J, Barron J, Achotegui V, Pijoan JI, Llorente JL (1999). Predisposing factors to bacterial colonization in chronic obstructive pulmonary disease. Eur Respir J.

